# Development and implementation of an LIS-based validation system for autoverification toward zero defects in the automated reporting of laboratory test results

**DOI:** 10.1186/s12911-021-01545-3

**Published:** 2021-06-02

**Authors:** Di Jin, Qing Wang, Dezhi Peng, Jiajia Wang, Bijuan Li, Yating Cheng, Nanxun Mo, Xiaoyan Deng, Ran Tao

**Affiliations:** 1 Laboratory Diagnosis Department, Jinan Kingmed Center for Clinical Laboratory, Jinan, 250100 China; 2 Clinical Laboratory Medicine, Guangzhou Medical University, Guangzhou, 510006 China; 3 Laboratory Diagnosis Department, Guangzhou Kingmed Center for Clinical Laboratory, Guangzhou, 510005 China

**Keywords:** Autoverification, Correctness verification, Integrity validation, Human–computer interaction, Risk control, Laboratory information system

## Abstract

**Background:**

Validation of the autoverification function is one of the critical steps to confirm its effectiveness before use. It is crucial to verify whether the programmed algorithm follows the expected logic and produces the expected results. This process has always relied on the assessment of human–machine consistency and is mostly a manually recorded and time-consuming activity with inherent subjectivity and arbitrariness that cannot guarantee a comprehensive, timely and continuous effectiveness evaluation of the autoverification function. To overcome these inherent limitations, we independently developed and implemented a laboratory information system (LIS)-based validation system for autoverification.

**Methods:**

We developed a correctness verification and integrity validation method (hereinafter referred to as the "new method") in the form of a human–machine dialog. The system records personnel review steps and determines whether the human–machine review results are consistent. Laboratory personnel then analyze the reasons for any inconsistency according to system prompts, add to or modify rules, reverify, and finally improve the accuracy of autoverification.

**Results:**

The validation system was successfully established and implemented. For a dataset consisting of 833 rules for 30 assays, 782 rules (93.87%) were successfully verified in the correctness verification phase, and 51 rules were deleted due to execution errors. In the integrity validation phase, 24 projects were easily verified, while the other 6 projects still required the additional rules or changes to the rule settings. Taking the Hepatitis B virus test as an example, from the setting of 65 rules to the automated releasing of 3000 reports, the validation time was reduced from 452 (manual verification) to 275 h (new method), a reduction in validation time of 177 h. Furthermore, 94.6% (168/182) of laboratory users believed the new method greatly reduced the workload, effectively controlled the report risk and felt satisfied. Since 2019, over 3.5 million reports have been automatically reviewed and issued without a single clinical complaint.

**Conclusion:**

To the best of our knowledge, this is the first report to realize autoverification validation as a human–machine interaction. The new method effectively controls the risks of autoverification, shortens time consumption, and improves the efficiency of laboratory verification.

## Background

Autoverification is a powerful tool for the batch processing of test results and has been widely used in recent years. It has obvious advantages in reducing reporting errors, shortening turnaround time and improving audit efficiency [1–5].

### Current status and challenges

Our self-developed autoverification system has been used for 6 years in many disciplines, such as biochemistry, immunology, hematology, microbiology, molecular analysis and pathology. To date, 25,487 rules have been set. The system judges test results 1.1 million times a day and provides audit recommendations for 250,000 report forms, accounting for 87% of the total number of report forms. Approximately 80,000 reports are automatically generated every day. To ensure the effectiveness and safety of the autoverification system, its validation process is very important. The College of American Pathologists clauses GEN43875 [6] and ISO 15189:2012 [7] 5.9.2b both require that autoverification systems undergo functional verification before use.

According to published studies, in laboratories that use autoverification, the majority of laboratories have performed personnel-based and automatic system audits with the same results, manually recorded consistency, and reached a conclusion after a statistical analysis of the results [2, 4, 8, 9]. The manual verification method is less difficult to operate but has the following limitations:

1. Massive validation workload. Based on the requirements of WS/T 616-2018 (China Health Organization recommended standard) [10] for validation of the autoverification of quantitative clinical laboratory test results, every test and every sample type involved in the autoverification procedure should be tested; the validation time should be no less than 3 months and/or the number of reports released should be no less than 50,000; and periodic verification should be performed every year for no less than 10 working days and/or for no less than 5000 reports. The validation workload is large, and it is difficult to rely on manual comparison and recording, which greatly increases the postanalytical workload.
2. Reporting risk [2]. During manual verification, personnel are prone to inertia or judgment errors. The lack of a system control mechanism for this kind of validation can generate reporting risks and directly affect clinical diagnosis and treatment.

Therefore, there is an urgent need to design a verification method that minimizes the workload and systematically controls risks. We report a rule verification system with a small workload and ease of operation that can be used as a reference for self-built and automatic test auditing for laboratories and manufacturers.

## Methods

### System design

Based on the American Clinical and Laboratory Standards Institute (CLSI) AUTO-10 [11] standards and current review processes, we established an autoverification system including 11 rule categories. Technicians set the rules according to audit requirements and rule categories. Each item can set multiple rules, including limited range check, combined mode judgment, Delta check, sampling time validity judgment, sample abnormality (hemolysis, lipemia) judgment and quality control check. The autoverification system determines whether the report is abnormal according to the rules. Tests that do not trigger contradiction mode are displayed in green, while failed tests (triggering rules, contradictory modes set by the rules) are displayed in red, and the cause of the contradiction is indicated. If all the tests in the report are green, the barcode of the report is also green. If any test in the report is red, the report shows a red barcode, which signals a warning in the system.

**Fig. 1:**
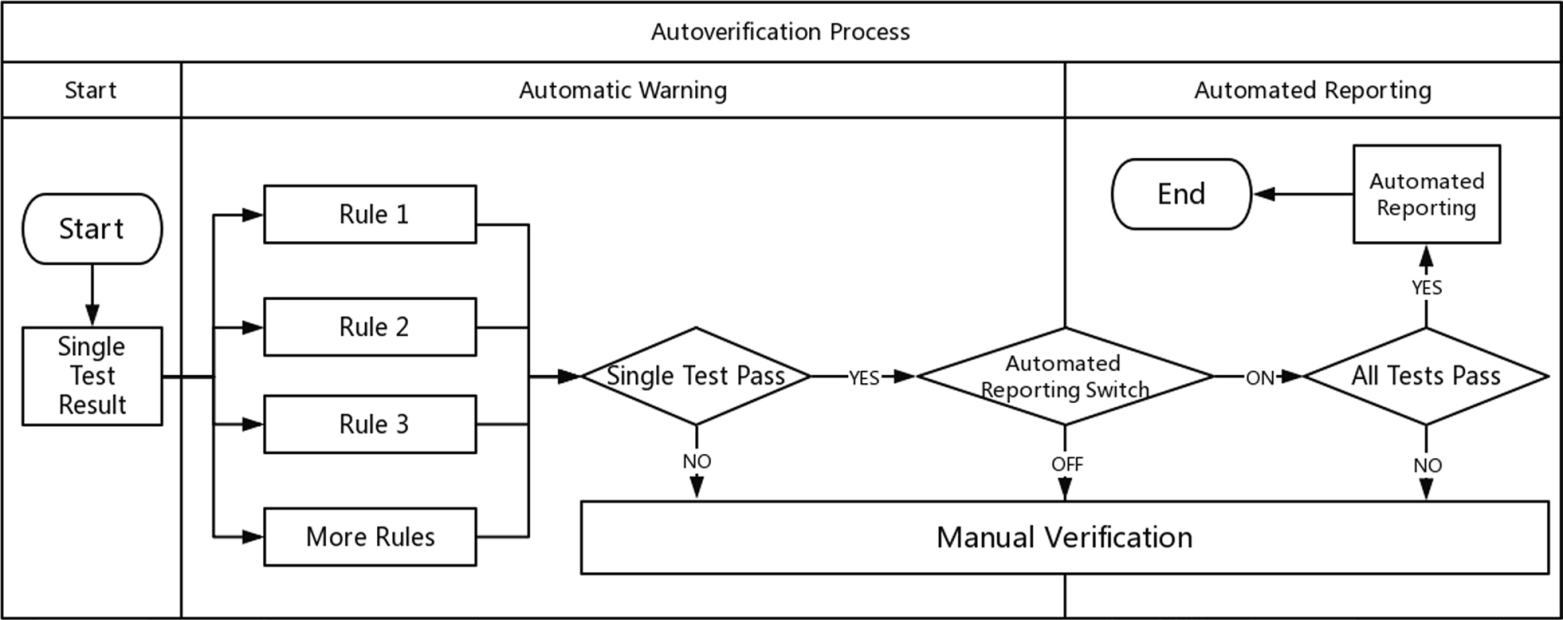
Autoverification process. Single test results must meet all the warning rules at the same time. The autoverification algorithm can identify those samples requiring manual review that do not meet the laboratory’s criteria for autoverification. If the automated reporting switch is not activated, then reports that pass the automatic warning step are manually issued. If the automated reporting switch is turned on and all tests on the report pass their warning rules, then the system automatically releases the report

According to the above steps, the autoverification system displays colors and abnormal prompts after judging the rules in a process called automatic early warning. The automatic warning is only for judgment and is not involved in the decision to issue a report. Based on this, the system automatically sends out a report with a green barcode in a process called automated reporting. Automatic early warning and automatic reporting comprise autoverification. This system is especially useful in the review of complex diagnostic projects (e.g., molecular testing, pathological testing). These projects prompt absurd values from personnel. For some moderately complex projects (e.g., biochemical, blood), the combination of report reviewing, automatic warning and automated reporting is equivalent to the autoverification system in a large number of literature reports and laboratory information system (LIS) automatic reports. The autoverification process used by our laboratory is shown in Fig. 1.

### Validation scheme

On the premise that automatic audits are divided into automatic warnings and automatic reports, we divide the verification system into two stages. The first stage is called correctness verification, which verifies that the operation of the rules is consistent with the expectations set by the personnel. If there is a problem, the responsible party may be the program development department. The second stage is called integrity validation. Based on the results from the first stage, this stage verifies whether the set rules include all the elements from the personnel’s audit report. The functional design of the two-stage system is shown in Table 1.

**Table 1:** Two validation methods designed for two parts of the autoverification system

Phase	Object	Validation method	Explanation	Inconsistent solutions
Automatic warning	Warning rules	Correctness verification	To verify that the warning rules behave as expected and produce the expected outcome	If the warning rule setting is wrong, delete and reset the rules
Automated reporting	Laboratory tests	Integrity validation	To confirm that the laboratory test results that pass the automatic warning can be reported automatically	Add more warning rules according to the laboratory report criteria

### Correctness verification

**Fig. 2:**
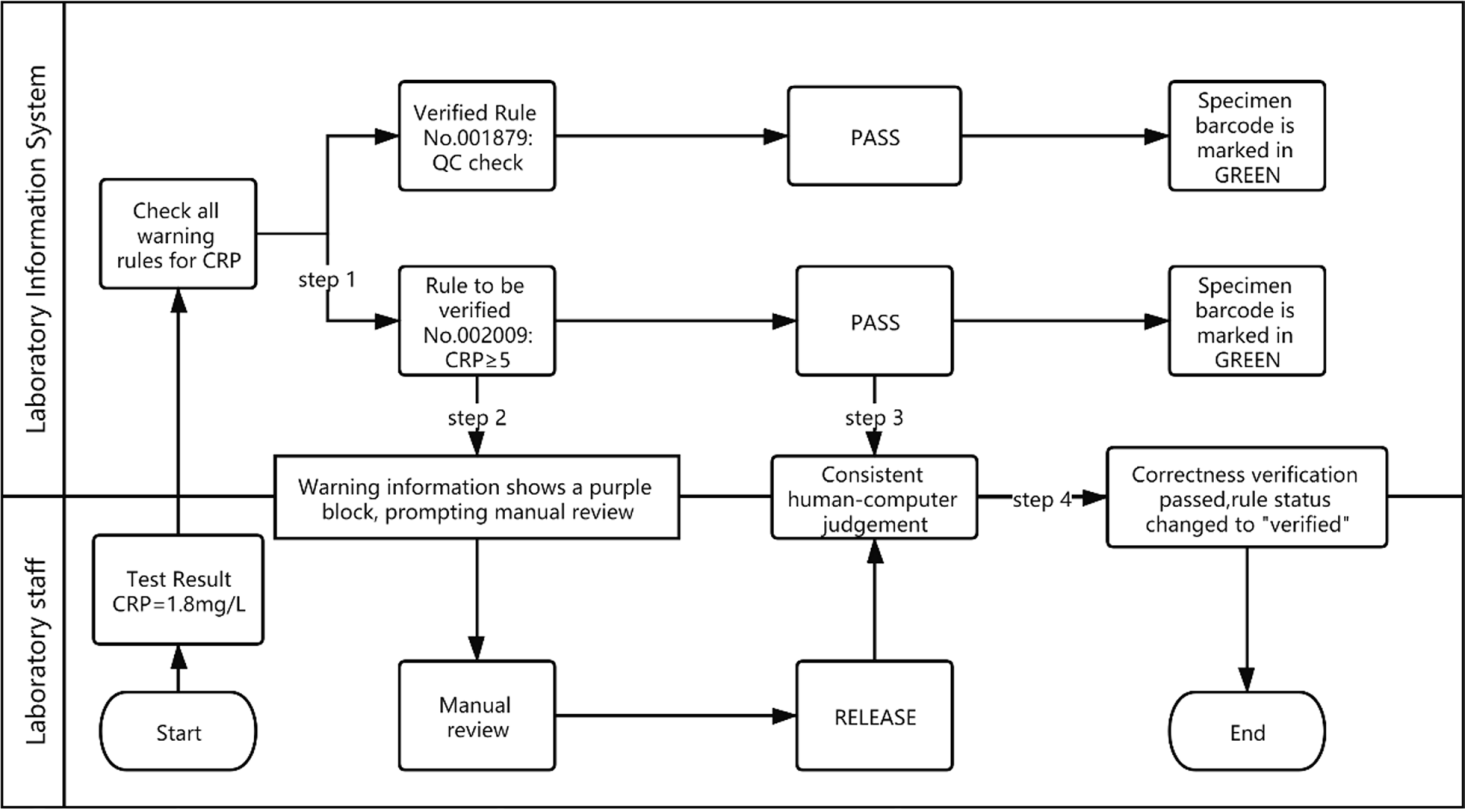
Schematic diagram of the correctness verification using the example of C-reactive protein (CRP). The CRP test result was 1.8 mg/l and passed quality control. The autoverification system searched all the rules for the CRP and hit two of them, No. 001879 and No. 002009. The No. 001879 rule (verified) checks whether the CRP result has passed the quality control. The No. 002009 rule (pending verification) intercepts the results greater than or equal to 5. Therefore, when No. 002009 is triggered, the warning information of the sample appears purple, indicating that the technician needs to confirm whether the warning result is consistent with the manual judgment. In the correctness verification interface as shown in Fig. 3, the system provides two options, the human–machine judgment is consistent or the system judges incorrectly. The technician can confirm that the rule is performing correctly and change its status to “verified”

The correctness verification phase confirms whether the execution of a single rule is correct. It is implemented as follows: (1) For newly added rules, the system adds the label "Pending Verification". (2) When the report is reviewed, the system displays the rule judgment result, and a purple color block is displayed to remind the staff to judge whether the execution result of the "Pending Verification" rule is correct. (3) The staff input the judgment result. (4) The system changes the rule status according to the staff input. If it is consistent, the rule label is set to "verified", prompting the personnel to continue to the next stage of verification. If it is inconsistent, the staff are prompted to delete the rule. Figure 2 is a schematic diagram of the correctness verification using the example of C-reactive protein (CRP).

**Fig. 3:**
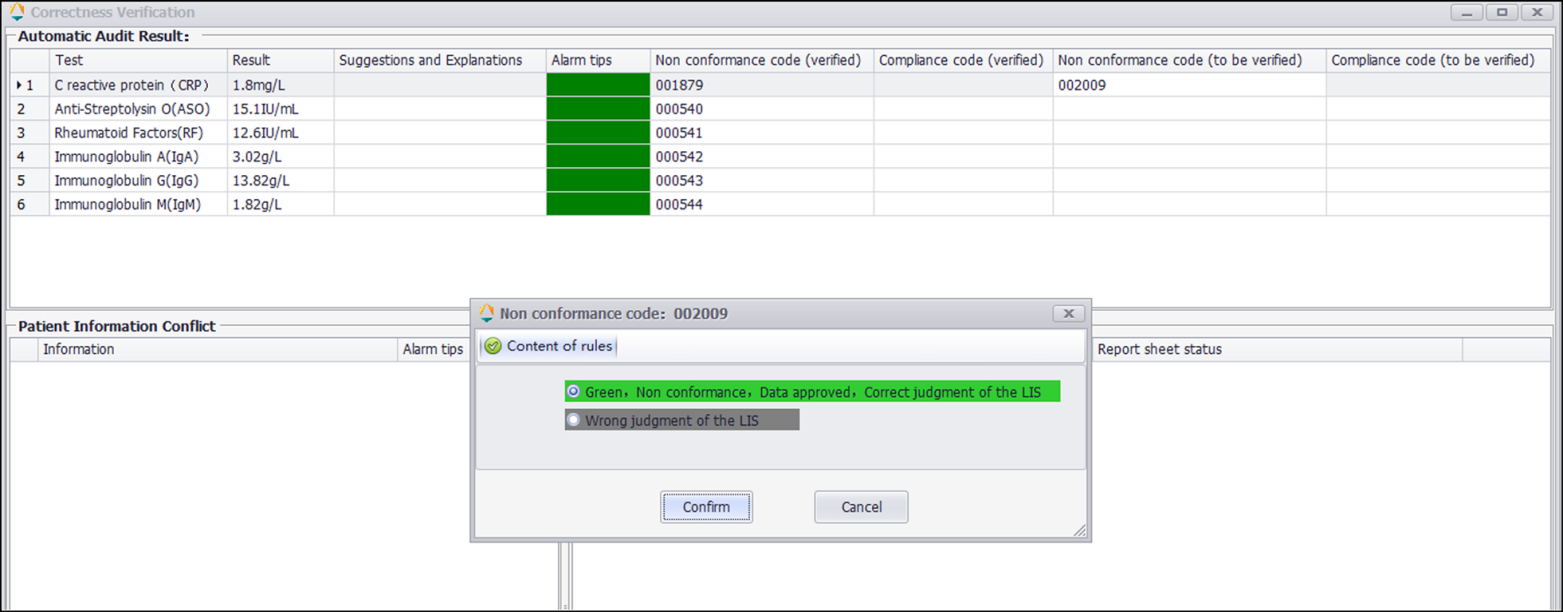
Correctness verification interface. The result of CRP passes automatic warning according to the No.002009 rule and displays green. The technician judges whether the automated warning operates correctly

### Integrity validation

**Fig. 4:**
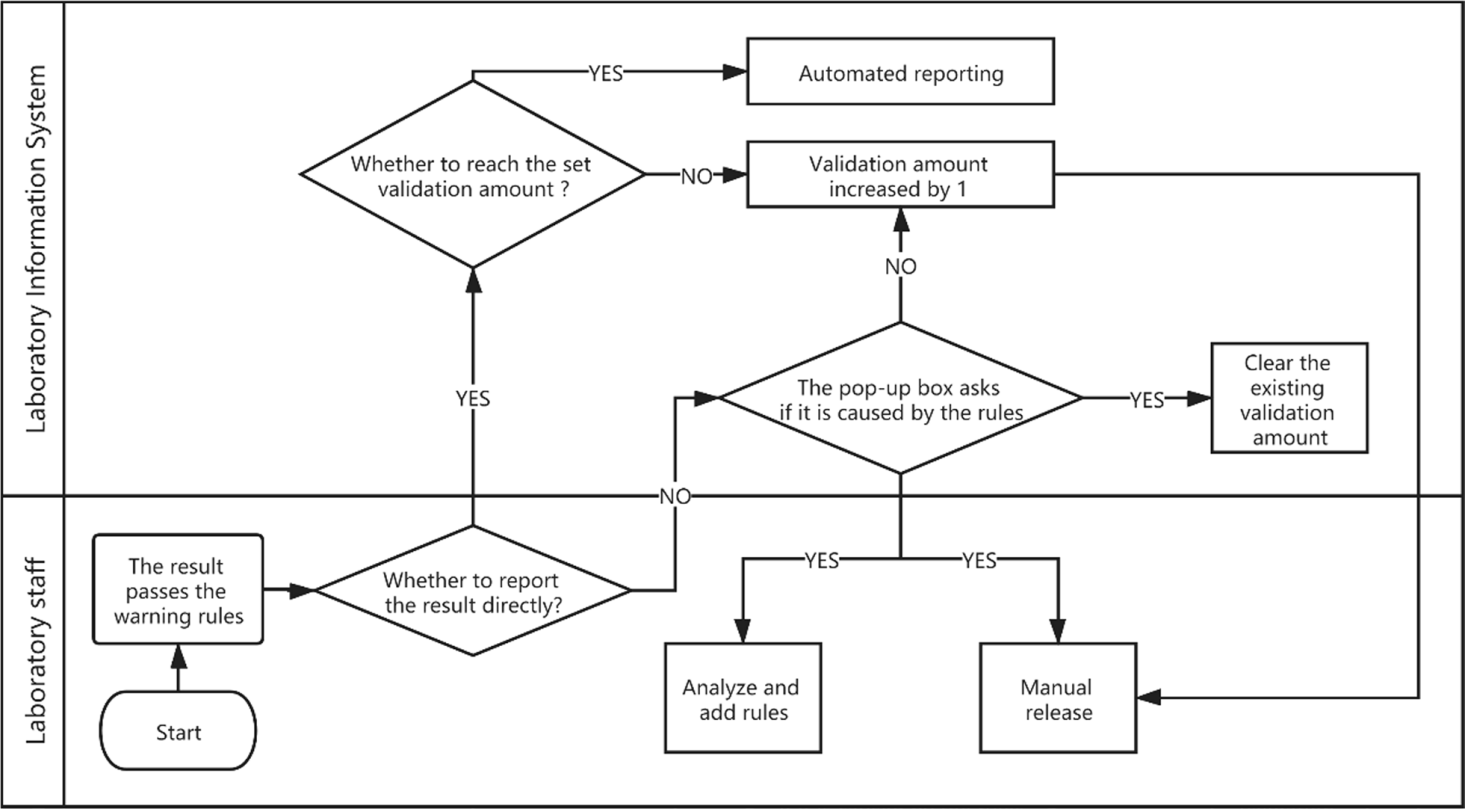
The integrity validation process

**Fig. 5:**
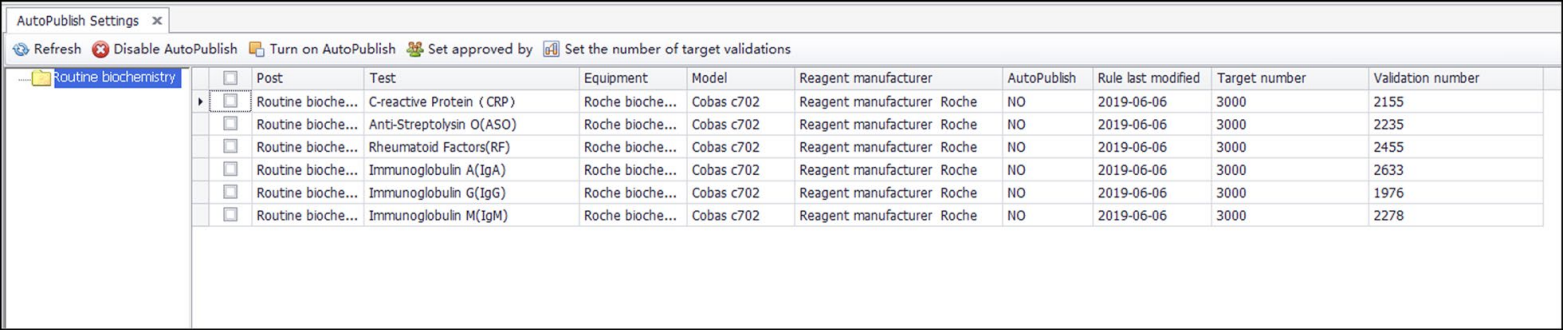
Integrity validation target number settings and recording interface. The validation targets of the six projects in the above figure are all 3000, and the validation number is between 1900 and 2500. The corresponding reports cannot be released automatically

Integrity validation can be started only after the correctness verification of all rules of a project is completed. It is implemented as follows. (1) After the report shows the result of the automatic warning, if the system detects that the report has been changed, a dialog box will pop up and ask the reviewer to select the reason for the modification. These reasons include (a) a rule execution error, (b) a rule setting value that is inappropriate, (c) the required addition of new rules, (d) the lack of involvement of other issues related to automatic review, and (e) automatic warning and prompt modification. The LIS records the modified content and the reasons for personnel analysis. (2) If the laboratory wants to implement automated reporting, a validation number, such as 5000, can be set according to the complexity of the project review. (3) If the automatic warning result of the report is green (approved), the personnel will issue the report directly, and the validation number of the report will automatically increase by one. (4) If the validation number of all items on the report exceeds the set number, the report will be automatically released. (5) If the automatic warning result of the report is green (approved), but the result is modified, with the reason for the modification specified as any of a, b or c, then the LIS will clear the validation number for the related items and stop automated reporting. Figure 4 shows the integrity validation process. The validation goals and validation amount for six projects are shown in Fig. 5.

### Accuracy guarantee

The accuracy of the autoverification includes whether the rules can be identified and whether there are omissions (completeness) in the report review. Therefore, our method confirms the accuracy of the new method from these two aspects. We perform function correction and system improvement through correctness verification and integrity validation. In the validation system, we design the following logic to ensure the accuracy of the function:

1. The new rule is automatically deleted if it fails the correctness verification within 10 days;
2. The rule is not allowed to be modified;
3. If the rule fails the correctness verification, it is forbidden to be converted;
4. If the autoverification of a single project fails the integrity validation, the historical validation amount is cleared.

### Data collection

The validation data of 30 assays from October 2019 to January 2020 were collected for analysis, and in total, 833 early warning rules were obtained. A total of 926,195 reports was used to evaluate the accuracy of the new method.

### Time consumption statistics

We used HBV as an example to introduce the comparison of the validation time before and after the new method was used. In the measurement of the validation time, we divided the complete autoverification into 10 stages. Time statistics were collected for manual verification and new method verification for each step. We used systematic records and estimates to develop time statistics for different stages.

### Satisfaction survey

We used questionnaires to evaluate the effectiveness of new methods used by laboratory technicians. The survey was launched using the online tool WJX (www.wjx.cn) which feeds back the percentage of responses and the total numbers.

## Results

### Correctness verification results

Among the 833 rules, 782 (93.88%) were successfully verified for correctness, with a total of 3814 validations, including 2230 (58.47%) released tests and 1584 (41.53%) intercepted tests. The inconsistencies were verified, and 51 (0.06%) error rules were deleted. The reasons for verification failure are shown in Table 2.

**Table 2:** List of reasons for correctness verification failure

Error type	Proportion (%)	Sample	Solution
Human error	63.3	Incorrect English letter case in the text of the rules, resulting in no warning	Reset the rules
Specific warning target	24.9	Early warning of diagnostic results and microscopy results in a special report interface for pathology	Add a supplementary algorithm code
Algorithm code error	8.4	HPV typing results could not be verified with the Delta Check; the results of the microbial project identification could not be correlated with a variety of drug sensitivity combinations	Fix the algorithm code
Software compatibility problem	3.4	Problem with the precision of the number comparison script	Fix the algorithm code

### Integrity validation results

We collected integrity validation data through system export and department feedback. The reasons provided for rule modification were automatic warning prompt and rule modification (5, 10.6%), rule execution error (0, 0%), improper setting values (15, 31.9%), new rule added (18, 38.3%), and no automatic warning involving other questions (9, 19.2%). The integrity of all projects was verified within 1 month, and the problems found are shown in Table 3.

**Table 3:** List of reasons why integrity validation failed

Test	Reason for not passing	Solution
HPV genotyping	There was no comprehensive analysis of the combined thin-layer cytology results	Analyze the results associated with thin-layer cytology
Urea	The limit range was too wide	Reduce the limit range
Albumin	Review of the detection system produces an error	Specify the detection system
CBC	Test results were checked only on the same day as the barcode	Extend the backdating of the historical results
HBsAg HBsAb HBeAg HBeAb HBcAb	Not all composite mode scenarios were covered	Add a joint audit of the portfolio project results
Cortisol	There was no warning of abnormal rhythms	Add a rule about checking sampling time

### Comparison of the two methods

The comparison of manual record analysis and the new method for different steps is shown in Table 4. The new method performs 4 automation steps, reduces the personnel workload, and automatically controls the enabling and disabling of automatic report release through system monitoring report modification. The increased accuracy verification can quickly eliminate rule setting exceptions and development loopholes while reducing the time needed for personnel analysis. The manual record analysis and the new method took 452 h and 275 h to complete, respectively.

**Table 4:** Comparison of the time consumption (hours) of the two methods for verifying HBV reports for 3000 cases

Steps	Manual validation (h)	New method (h)
1. Set 65 rules	1.5	1.5
2. Perform Rule 130 test	2.5	2.5
3. Correctness verification	0	0.25^a^
4. Personnel comparison report and results review	240	240
5. Record comparison result	100	0^b^
6. Analysis of the verification number	10	0^b^
7. Determine whether to activate automatic approval	5	0^b^
8. Personnel analysis of the reasons for inconsistent audit results	90	30
9. Add and modify rules	1	1
10. Determine whether to turn off autoverification	1	0^c^
Total	452	275

In the measurement of the validation time, we divided the complete autoverification into 10 stages. The statistics of manual verification and the new method for each step are shown in Table 4. In steps 4–6, in total, 3000 reports are used for statistics. The time consumption of the consistent work content in the new and old methods is subject to the following: the manual timing of the old method, such as steps 1, 2, 4, and 9; the inconsistent steps in the two methods; the new steps that are recorded in the system, such as step 3; the saving step time clearing, such as steps 5, 6, 7, and 10; and the remaining steps that are estimated, such as step 8

For automatic implementation, the time is calculated as zero

^a^Reasons for invalid locking rules

^b^Reduced workload

^c^Controlled risks

### Satisfaction survey

After using the new method for 1 year, we conducted a satisfaction survey of laboratory personnel who used the function, distributed 182 questionnaires, and recovered 168 copies, with a response rate of 92.3%. The survey results showed that 94.6% of laboratory users believed that the new method could greatly reduce the workload, effectively control the report risk, and produce satisfactory or very satisfactory assessments of the new method.

## Discussion

The core of the use of autoverification lies in the validation of system functions and rules. Due to the complexity of these rules, it is impossible to find all the functional defects by relying solely on function validation before the system goes online, and even human input errors cannot be carried out in the validation [12]. Such functional defects must be found in actual application scenarios with multiple different rule settings, such as the incorrect input of full-width symbols, that is, correctness verification. Furthermore, the premise of rule verification is to include a review of the logic of all reviewers in the system, which can be discovered only in actual application scenarios. Additionally, integrity validation can be performed in actual application scenarios to truly find problems [13, 14].

We initially designed the system in two parts, automatic warning and automated reporting, to allow complex detection items (molecular and pathological examinations, final human reports, and system prompt errors) to be included in the automatic review. Laboratory technicians can then choose to address the needs of different measurements. These two parts correspond to two verification steps: the automatic warning part performs correctness verification, and the automated reporting part performs integrity validation.

**Fig. 6:**
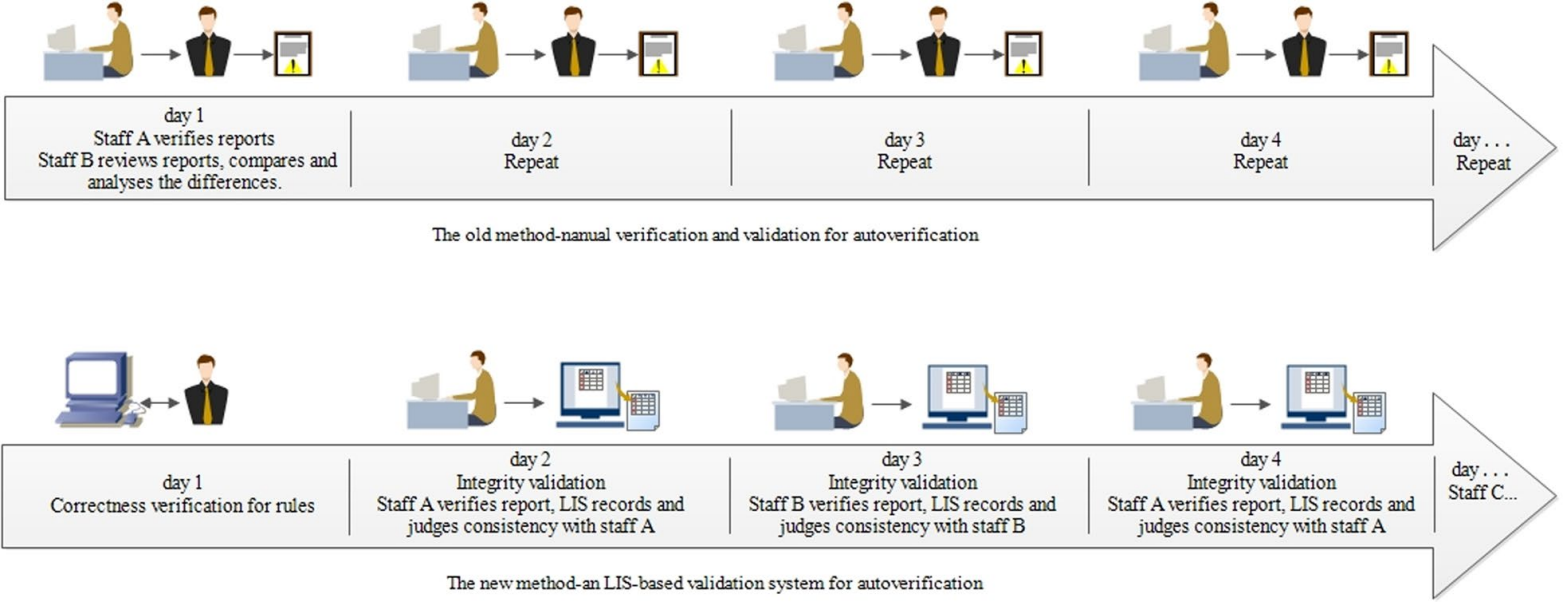
Schematic diagram of the process comparison between the manual method and the new method

Compared with other systems reported in the literature [4], the advantages of the new method are mainly simplifying the verification process, reducing the verification workload and ensuring the accuracy of the verification results. As shown in Fig. 6, in the manual verification scheme, the junior staff will review the results, and then the intermediate staff will repeat reviewing the results and combine the autoverification results to determine the human–machine consistency to complete the autoverification function validation. The whole process focuses on human leadership. The new method uses system monitoring to judge the accuracy of the autoverification based on the operational trajectories of different personnel. By interacting with personnel, the system collects validation data and controls the operation of autoverification.

We delineate the advantages of the new method compared to manual validation in Table 5.

**Table 5:** Comparison of the advantages of the new method and manual verification

Advantages	Difference	Manual validation	New method	Explanation
Efficiency improvement	Whether to add extra workload?	YES	NO	No additional personnel are required to manually record the reason for the inconsistency. The new method is that the system completes judgment and records while personnel review the reports normally. The system will control the operation of the autoverification program based on the consistency results
	Can the cause of inconsistency be quickly determined?	NO	YES	The main reasons for the inconsistency are abnormal rule settings and lack of necessary rules. The new method correspondingly sets up correctness verification and integrity validation for these two main reasons. In different verification stages, only the main reason for that stage can be traced back
Risk control	Is it possible to skip the validation process?	YES	NO	Starting from setting the rules, the system will pull the validation process, and no validation link can be skipped
	Whether to ensure sufficient amount of validation data?	NO	YES	In the process of normal personnel issuance, the system will truthfully record the validation data. Before the set data volume is reached, the automated reporting function is prohibited
	Can autoverification be used in the case of failed validation?	YES	NO	When the system confirms that the validation fails due to a defect in the autoverification, it will prohibit the rule conversion or the automated reporting from being enabled

Compared with the traditional method, the true positives and false positives of the "personal and machine-based audit results" are easy to understand, but if the indicators are abnormal, it can be difficult to find the cause of this abnormality, especially after all the reasons are verified after thousands of reports are released [15]. Consequently, the audit scenario has become blurred in the auditor's memory, and it becomes inefficient to check the problems one by one. The process-based validation scheme that we developed is more practical and advantageous: (1) It can be easily operated and quickly initialized; (2) its self-traction and control of online functions can ensure that every rule is fully verified; and (3) the amount of manual work is small, allowing technicians to complete the verification steps during their daily work.

We divided the entire validation into two modules, correctness verification and integrity validation, based on the concept of process management. Rules are the basic unit of the entire autoverification system. If basic rule verification is not performed at the beginning of the entire process, when the human–machine judgment is inconsistent, it is difficult to confirm whether the problem is caused by algorithm error, execution error or another reason, inevitably increasing the analysis workload. In contrast, if correctness verification is completed when the rules are established, the only reason for an inconsistency between man and machine during the release of the report issuance would be "rule omission", requiring the technician to add only the corresponding rules.

During the entire verification process, we implemented human–computer interaction, which includes the following:

1. An "expected sense of play": Before the laboratory personnel view the results, they already possess a logical expectation, and in the process, they establish a comparison of the rules and effects;
2. The use of visual stimulation methods (red, green, and purple backgrounds) that can be quickly identified and relax the laboratory personnel; and
3. System pull—once the verification succeeds or fails, it is automatically counted with the click of a button, which automatically opens the automatic report function. All the functions ensure that laboratory personnel, particularly those of the new generation, can derive enjoyment from completing the verification process, thus increasing its core value [16].

According to the experience of this research, the logic of the autoverification validation process is not difficult, but if it is applied to other laboratories on a large scale, the intermediate software supplier needs to develop the original autoverification system. The validation system is based on the autoverification system developed by our laboratory, so it is more compatible in adding new functions. However, as a supplementary function, it is difficult to graft to existing systems. We suggest that peers can refer to the program logic provided in this study. On the basis of the current functions, we will further strengthen the learning ability of the validation system and convert validation records into learning cases that can serve as a guide for laboratory technicians to use the autoverification function more efficiently.

## Conclusions

In the 2 years that the online validation has been in use, there have never been any defects or reporting risks due to autoverification. We believe that for both intermediate and self-built autoverification systems, online validation is a useful tool for controlling the risks of autoverification and improving the quality of reports. The detailed process for this method can serve as reference for the development and implementation of LIS-based autoverification systems.

## Data Availability

All data generated or analyzed during this study are included in this published article. The data underlying this study are available and researchers may submit data requests to the corresponding author on reasonable request.

## Abbreviations

LIS: Laboratory information system
TAT: Turnaround time
CLSI: American Institute of Clinical and Laboratory Standardization
AUTO 10-A: Autoverification of Clinical Laboratory Test Result 10-A; Approved Guideline
CRP: C-reactive protein
CBC: Complete blood cell count
HBsAg: Hepatitis B virus surface antigen
HBsAb: Hepatitis B virus surface antibody
HBeAg: Hepatitis B virus e antigen
HBeAb: Hepatitis B virus e antibody
HBcAb: Hepatitis B virus core antibody
HBV: Hepatitis B virus
HPV: Human papilloma virus
